# Surface-spreading technique of meiotic cells and immunodetection of synaptonemal complex proteins in teleostean fishes

**DOI:** 10.1186/s13039-015-0108-9

**Published:** 2015-01-27

**Authors:** Cristian Araya-Jaime, Érica Alves Serrano, Duílio Mazzoni Zerbinato de Andrade Silva, Masakane Yamashita, Toshiharu Iwai, Cláudio Oliveira, Fausto Foresti

**Affiliations:** Departamento de Morfologia, Instituto de Biociências, Universidade Estadual Paulista, Distrito de Rubião Junior, s/n, 18618-970 Botucatu, SP Brazil; Department of Biological Sciences, Faculty of Science Hokkaido University, Sapporo, 060-0810 Japan; South Ehime Fisheries Research Center, Ehime University, Matsuyama, Ehime 798-4292 Japan

**Keywords:** Meiosis, SYCP3, Method, Co-detection, FISH

## Abstract

**Background:**

Different moderrn methodologies are presently available to analyze meiotic chromosomes. These methods permit investigation of the behavior of chromosomes in the normal complement and of sex and B chromosomes, two special types of chromosomes that are associated with the A complement and are present in many organisms, including fishes. However, meiotic studies are still scarce in fishes, considering the wide number of species in this group.. Here, we describe a new protocol for the visualization of the synaptonemal complex in spermatocytes and oocytes of fishes and to the sequential use of the technique with other procedures and techniques such as immunodetection of the synaptonemal complex protein with a specific antibody and co-detection of DNA sequences by FISH.

**Results:**

The meiotic surface-spreading protocol used in the present proposal worked well in representative species of four fish orders and was useful in obtaining good results even in small specimens. Fish-specific antibodies and commercial products worked similarly well to detect synaptonemal complex (SC) proteins. The sequential application of fluorescence *in situ* hybridization using specific probes showed clear signals associated with the SC structures identified by immunostaining.

**Conclusion:**

Here, we provide a useful and applicable immunofluorescent protocol for the visualization of synaptonemal complex proteins in the meiotic cells of fishes in surface-spreading preparations. Furthermore, this technique allows for the sequential application of other cytogenetic procedures.

## Background

During meiosis, specific events are required to promote genetic diversity and ensure the segregation of homologous chromosomes. These events, including synapsis and meiotic recombination between homologous chromosomes, cause sequential structural changes in chromosomes [1]. Thus, studies on these structural elements can provide important information about pairing and synapsis processes involving homologous chromosomes [2], as well as about the evolution of protein components of the synaptonemal complex (SC) among vertebrates [3].

To accomplish reductional segregation, homologous chromosomes must recognize one another and identify their partners, forming a meiosis-specific organizational structure known as the SC [4,5], which is involved in the chromosome pairing process.

The SC consists of three basic structural filamentous proteins types: two axial or lateral elements connected to the chromatin fibers of chromosomes, one central layer element, and transverse filaments that connect the central layer and the lateral elements [6]. In mammals, seven proteins are described as part of the SC: SYCP2 and SYCP3, forming the lateral elements [7,8]; SYCP1, which is the unique protein that joins the transversal elements [9] and four small proteins, SYCE1, SYCE2, SYCE3 and Tex12, which are specific to the central element [10–12].

In fishes, the presence of a variety of different chromosome polymorphism types, including sex chromosomes and supernumerary chromosomes, has sparked interest in finding information that could lead to a better understanding of the evolutionary processes related to the origin and structure of these chromosomes. However, studies that assess the structure or behavior of chromosomes during meiosis are restricted to a few species, including *Danio rerio* [13], *Acipenser transmontanus* [14], *Oryzias latipes* [15] and *Oncorhynchus mykiss* [16]. In some works, meiotic chromosomes were analyzed either to compare the pairing processes of chromosomes between closely related species [17,18] or to identify and study the evolution, behavior and origin of sex chromosomes [16,17,19] and B chromosomes [20,21]. The methodology applied in these studies can be difficult to transfer and use in certain groups of fishes in which the pachytene stage occurs in the early phase of the development [4]; in such fishes, the specimens are too small to provide reasonable amounts of tissue to be studied. Another limitation in the application of the technique is the necessity of using electron microscopy to visualize the SC, which is a laborious task and frequently makes it difficult to obtain viable and clear results. Finally, the techniques are not appropriate or not designed to be sequentially used with other cytogenetic procedures.

Here, we describe a new and efficient protocol for the visualization of the SC in spermatocytes and oocytes of fishes adapted from the protocols described by Van Eenennaam *et al*. [14] and Campos-Ramos *et al*. [19]. We also demonstrate that the new methodproposed here is effective for species of several groups and even for use in combination with other methods, such as immunodetection of the SYCP3 protein with a specific antibody and co-detection of DNA sequences by fluorescence *in situ* hybridization (FISH).

## Results

**Figure 1:**
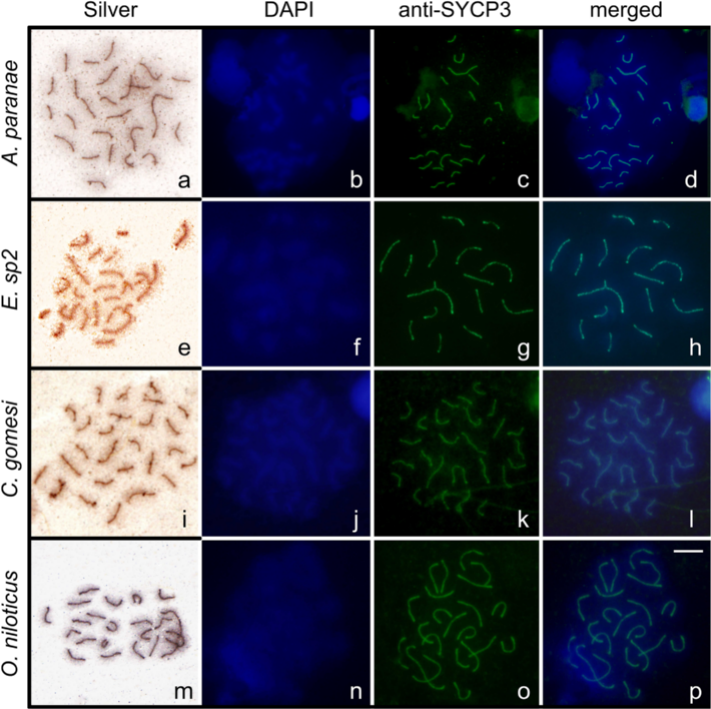
**Synaptonemal complex detection with silver stain and with anti-medaka SYCP3 antibody.** Meiotic nuclear microspreads in oocytes of *Astyanax paranae* **(a, b, c, d)**. Spermatocytes of *Eigenmannia* sp2 **(e, f, g, h)**, *Characidium gomesi* **(i, j, k, l)** and *Oreochromis niloticus* **(m, n, o, p)**. spermatocytes and oocytes were conterstained by DAPI. Bar = 10 μm.

**Figure 2:**
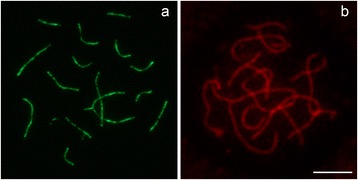
**Immunodetection in** ***Eigenmannia*** **sp2 spermatocytes using commercial antibodies against mouse and rabbit SYCP3 (Abcam ab97672). a)** anti-mouse SYCP3 primary antibody/FITC-labeled secondary antibody and **b)** anti-rabbit SYCP3 primary antibody/Texas Red-labeled secondary antibody. Bar = 10 μm.

Meiotic preparations from different species of fishes such as *Oreochromis niloticus*, *Eigenmannia* sp2, *Astyanax paranae*, and *Characidium gomesi* were obtained with application of the surface-spreading protocol proposed in this study (Figure 1), highlighting the replicability of the method. Additionally, the results of SYCP3 SC protein immunodetection using a fish-specific antibody were similar to those obtained using mammalian antibodies (Figure 2).

**Figure 3:**
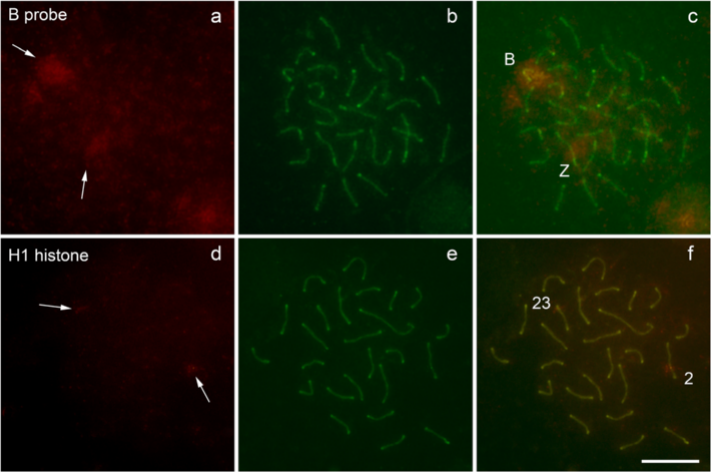
**FISH hybridization after immunodetection of the synaptonemal complex with anti-medaka SYCP3 antibody.** Chromosome painting with specific probes for B chromosome in *Characidium gomesi* **(a, b, c)** and histone H1 probe hybridization in *Astyanax paranae* **(d, e, f)**. In c and f, FISH hybridization signs are visualized on the specific bivalent chromosomes. Bar = 10 μm.

Sequential detection of specific DNA sequences encoding histone H1 by FISH on surface-spreading preparations for SC identification showed clear signals associated with the SC complexes (Figure 3a,b,c), on sites corresponding to specific bivalents. Similarly, chromosome painting with a B chromosome probe revealed the presence of fluorescent signals associated with the SC complex corresponding to this chromosome (Figure 3d,e,f).

## Discussion

The versatility and repeatability of this method coupled with the applicability of this method with complementary cytogenetic techniques greatly increased the resolution capability of the technique to study the composition, structure, and behavior of meiotic chromosomes. The protocol described here allows for the use of fish-specific or commercial, mammalian-derived antibodies to detect protein components of the SC in fishes following pretreatment with citrate buffer, which exposes the protein complexes and allows for antibody binding. The ability to use either fish-specific or mammalian-derived antibodies highlights the state of meiotic protein conservation among vertebrates [15,20]. The preparations obtained using this protocol can also be sequentially used for other techniques such as silver nitrate staining, immunostaining, and fluorescence *in situ* hybridization to identify the localization of DNA markers. The use of FISH combined with immunostaining in these preparations allows for simultaneous visualization of the pairing behavior of bivalent chromosomes, unpaired regions, and the location of molecular markers or the painting of whole chromosomes during the pachytene stage [21]. Remarkably, this technique is also very useful for small specimens because it can be adapted for the specific volume of the cell suspension for each specimen; further, the number of slides per volume of cell suspension can be altered.

To study meiosis in organisms in the pachytene stage, knowledge of the exact sexual maturation phase of the organisms is essentially required for obtaining good SC preparations in males and females. The pachytene stage occurs in the initial reproductive steps of some species and can be visualized in the early stages of gonadal development [22,23]. Some species are generally too small in this stage, leading to difficulty obtaining a reasonable amount of tissue [24–26]. In fishes, some species breed throughout the year, while others breed only once per year or at certain times during the year, and environmental changes can directly influence the period of gonadal maturation [27–30]. Thus, the main challenge this method serves to successfully address is to provide a perfect understanding of the timing of the meiotic cycle of these species.

## Conclusions

The results reported here demonstrate the repeatability and versatility of the protocol for immunodetection of SC proteins in meiotic surface-spreading preparations of fishes. The possibility of using this SC immunodetection technique with the sequential application of other cytogenetic methodologies, including FISH using DNA probes for specific sequences or entire chromosomes, can enhance the resolution of fishes meiotic studies and make possible the identification of chromosomal segments or entire chromosomes. Thus, this method improves our ability to analyze the meiotic behavior of sex chromosomes, B chromosomes and specific chromosome sites during the early stages of meiosis.

## Methods

### Animal Sampling

This protocol was tested in five fishes species representing the orders Perciformes (*Oreochromis niloticus*), Gymnotiformes (*Eigenmannia sp1, Eigenmannia* sp2) and Characiformes (*Astyanax paranae* and *Characidium gomesi*).

#### Surface-spreading protocol

*Protocol*

- Sacrifice individuals with an overdose of benzocaine.
- Remove the gonads (testes or ovaries) and immerse them in 200 μl cold Hank’s solution (Sigma) in a Petri dish. The volume of saline solution should represent threefold the tissue volume.
- Mince tissue to obtain a fine cell suspension.
- Transfer cell suspension to a 1.5 ml tube and decant for 30 min.
- Place a drop of cell suspension (approximately 20 μl) in the middle of a clean, dry slide, avoiding the presence of cell debris.
- Add 2 drops of 0.2 M sucrose and 2 drops of 0.2% Triton X-100 onto the cells; all the solutions must be adjusted to pH 8.5 with 0.1 M sodium tetraborate.
- Incubate the preparations containing the testis cell or ovarian cell suspensions for 4 min or 7 min, respectively.
- Fix the cells on the slides by adding 10 drops of 4% paraformaldehyde solution (pH 8.5, adjusted with 0.1 M sodium tetraborate) for 15 min.
- Remove excess liquid with absorbent paper and let dry at room temperature for 3 hr.
- Wash 3 times with 0.08% Photo Flo 200 (Kodak) solution and dry in a vertical position.
- Assess the quality of the preparations by silver nitrate staining according to the protocol described by Howell & Black [31].
- Store preparations at -20°C in the deep freezer.

#### Immunostaining

- Wash the slides three times in 1× PBS for 5 min; do not allow the slides to dry.
- Antigen recovery*: immerse the slides in 0.01 M citrate buffer (pH 6), preheated to 95°C for 20 min.
- Allow cooling at room temperature for 20 min.
- Wash three times in 1× PBS for 2 min each.
- Incubate with primary antibody (1/100 in 1× PBS), add 25 μl per slide, and cover with a coverslip for 1.5 hr at 37°C in a humid chamber*.
- Wash three times in 1× PBS for 5 min each (room temperature).
- Incubate with secondary antibody (1/100 in 1× PBS) by adding 25 μl per slide with a coverslip for 40 min at 37°C.
- Wash three times 1× PBS, for 5 min each.
- Mount preparations under coverslips with 15 μL. Vectashield/DAPI.

* The time and temperature for primary antibody incubation can be modified, using either 3 hr. at room temperature or overnight at 4°C.

#### Sequential FISH and chromosome painting

Following immunodetection, preparations were submitted to FISH. However, as FISH can dim the immunodetection signal, images were taken before this next step was conducted, to be combined with the final image after FISH. The final image composition was performed using image editor software with the double-FISH images, using a uniform image scale and size. However, the SC detection can be improved during FISH by adding secondary antibody to the hybridization solution.

Probes for histone H1 gene and chromosome painting for B chromosomes were produced and labeled according to Hashimoto *et al.* [32] and de Silva *et al.* [33] respectively. FISH was performed under highly stringent conditions using the method described by Pinkel et al. [34], without pretreatment steps. Initially, the slides were washed twince in 2× SSC to remove DAPI (4’, 6-diamidino-2-phenylindole, Vector Laboratories). The chromosomal DNA was denatured in 70% formamide/2× SSC for 5 min at 70°C. For each slide, 30 μl of hybridization solution containing 200 ng of each labeled probe, 50% formamide, 2× SSC and 10% dextran sulfate was denatured for 10 min at 95°C, dropped on the slides and hybridized overnight at 37°C in a 2× SSC moist chamber. Post hybridization, the slides were washed two times in 0.2× SSC/15% formamide for 10 min at 42°C, followed by a second wash three times in 0.1× SSC for 5 min at 60°C and a final wash at room temperature in 4× SSC/0.5% Tween for 5 min. Probe detection was carried out with Avidin-FITC (Sigma) or anti-digoxigenin-rhodamine (Roche) and chromosomes were counterstained with DAPI.

## Endnotes

Antigen recovery*: Following pretreatment with citrate buffer, the binding sites for the complex of proteins became most exposed for antibody binding.
